# Supplementary material for: Occurrence and repair of alkylating stress in the intracellular pathogen Brucella abortus
Source: Nat Commun. 2019 Oct 24;10:4847. doi: 10.1038/s41467-019-12516-8 (PMC6813329; doi:10.1038/s41467-019-12516-8)
Supplement: Supplementary file 1 — Supplementary Information [file 41467_2019_12516_MOESM1_ESM.pdf]

Supplementary Information

Occurrence and repair of alkylating stress in the intracellular pathogen *Brucella abortus*

Katy Poncin[1,2], Agnès Roba[1], Ravikumar Jimmidi[3], Georges Potemberg[1], Antonella Fioravanti[4,5], Nayla Francis[1], Kévin Willemart[1], Nicolas Zeippen[1], Arnaud Machelart[1,6], Emanuele G. Biondi[4], Eric Muraille[7], Stéphane P. Vincent[2], Xavier De Bolle[1*]

[1] URBM, University of Namur, Namur, Belgium. [2] Sir William Dunn School of Pathology, University of Oxford, Sir Parks Road, Oxford OX1 3RE, UK. [3] Unité de Chimie Organique, University of Namur, Namur, Belgium. [4] Unité de Glycobiologie Structurale et Fonctionnelle, UMR 8576 CNRS, Université de Lille, 50 Avenue Halley, Villeneuve d'Ascq, France. [5] VIB ,Vrije Universiteit Brussel, Brussels, Belgium. [6] Université de Lille, CNRS, INSERM, CHU Lille, Institut Pasteur de Lille, U1019, UMR 8204, Center for Infection and Immunity of Lille, Lille, France. [7] IMM, Aix-Marseille Université, Marseille, France. [8] Laboratoire de Parasitologie, Faculté de Médecine, Université Libre de Bruxelles, Brussels, Belgium.
*Corresponding author: Xavier De Bolle (xavier.debolle@unamur.be)

Supplementary Figure 1

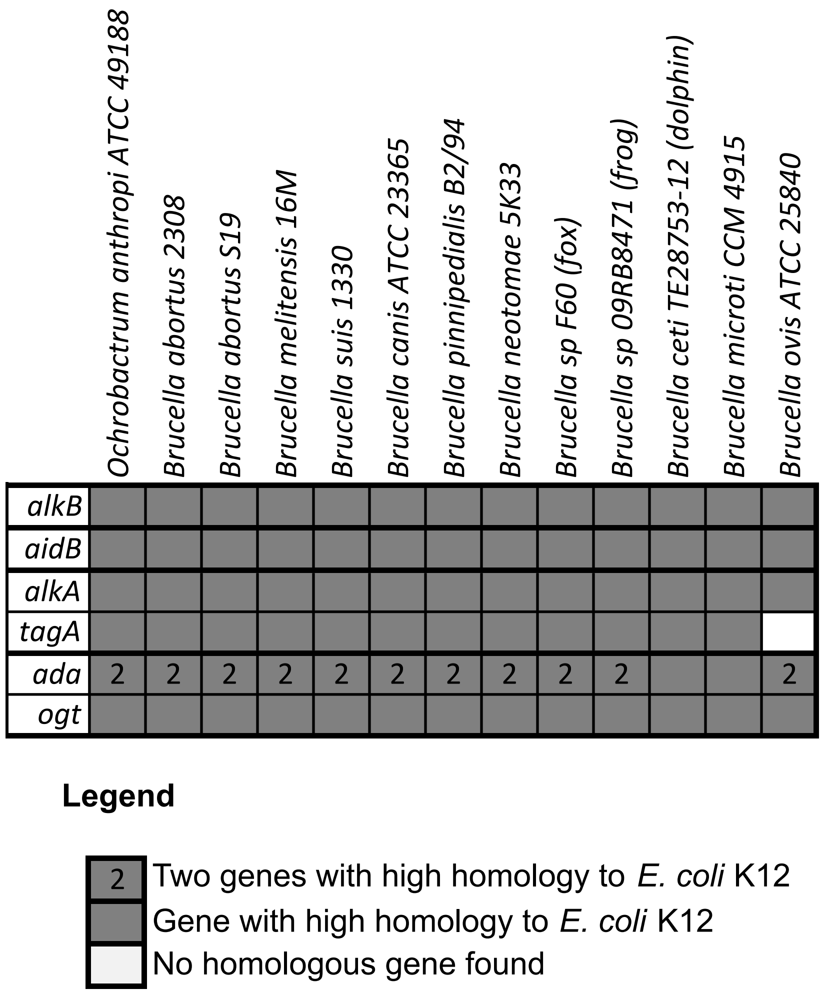

Supplementary Figure 1. Conservation of genes coding for alkylated DNA repair proteins in *Brucella* species and a close relative, *Ochrobactrum anthropi*. Genes were grouped by function. In the case of *B. ceti* and *B. microti*, two distinct homologues of *ada* and *ogt* were also found but not included in the figure. Homology was calculated based on *E. coli* K12 genome (www.patricbrc.org).

Supplementary Figure 2

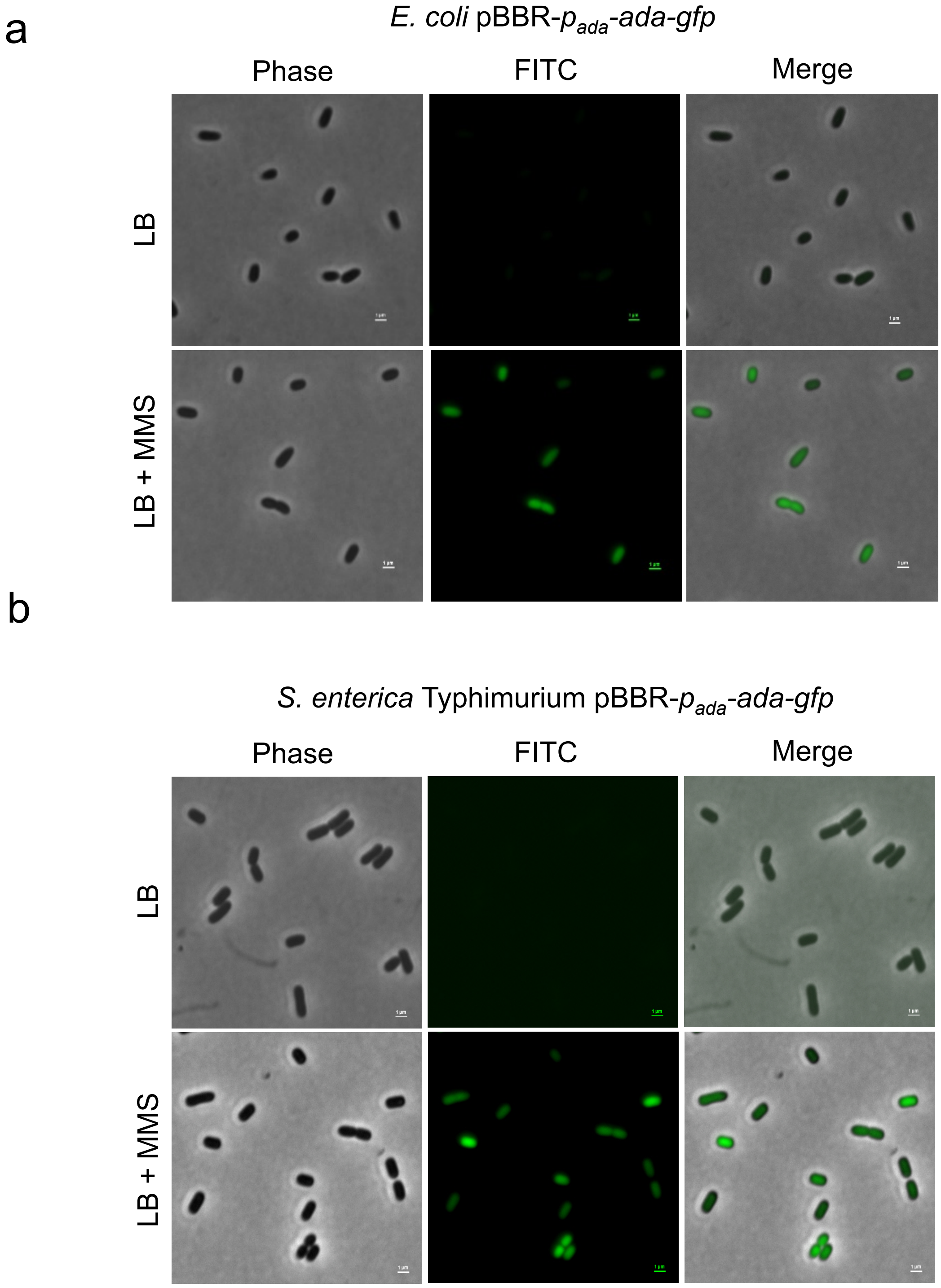

Supplementary Figure 2. Reporter system in **a.** *E. coli* and **b.** *S. enterica* Typhimurium. The reporter system to detect alkylating stress was used in both bacteria cultured for 1h45 in the absence of stress (LB only) or in the presence of an alkylating agent (LB supplemented with 0.5 and 1 mM of MMS, respectively). Scale bars represent 1 µm.

Supplementary Figure 3

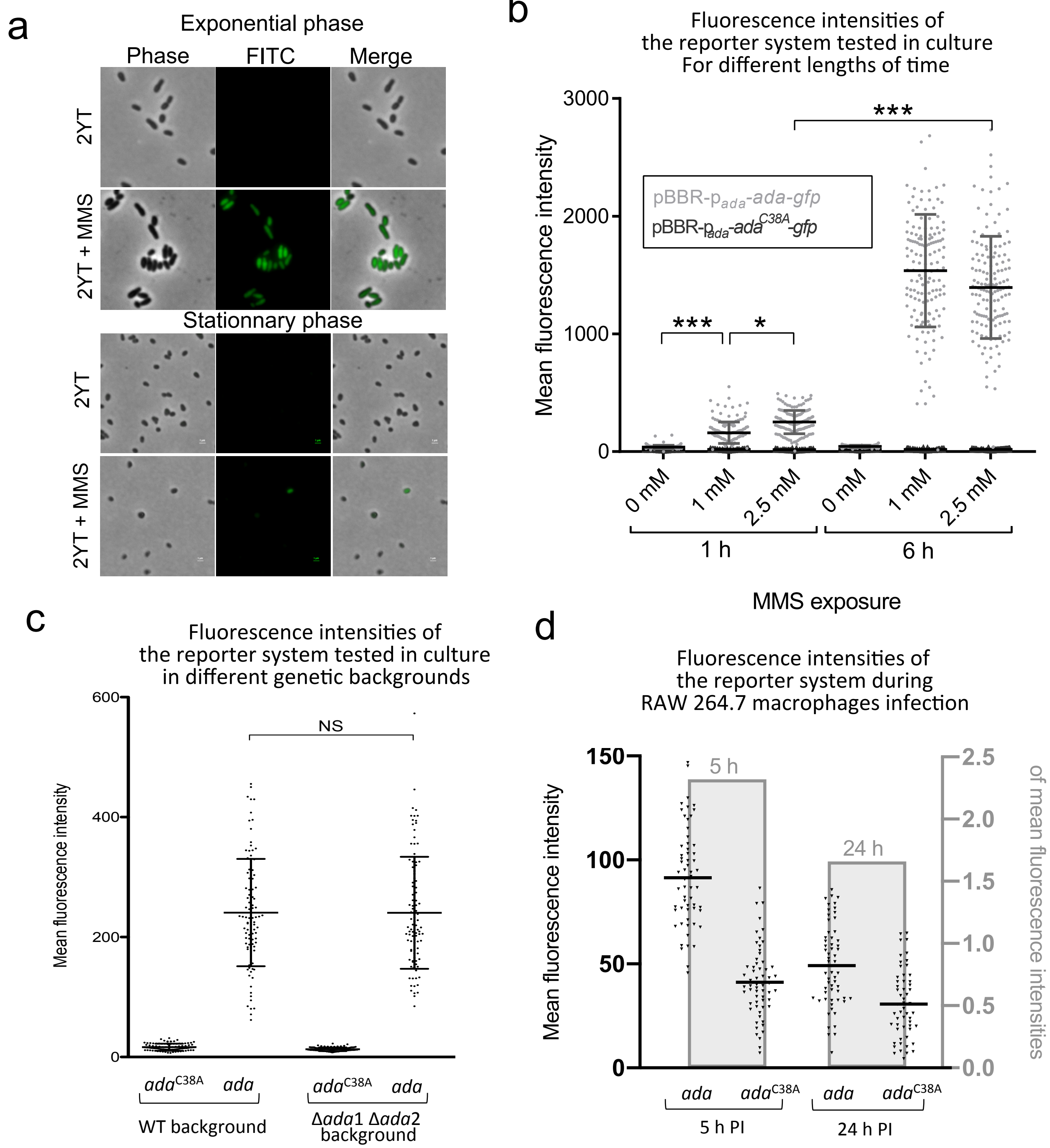

Supplementary Figure 3. Reporter system for alkylating stress tested on *B. abortus*. a. *B. abortus* carrying the pBBR-*p*$_{ada}$-*ada-gfp* reporter system was cultured to reach either exponential phase (OD$_{600}$ 0.6) or stationary phase (OD$_{600}$ 1.2) in rich liquid medium (2YT), then it was exposed to 5 mM of MMS for 1h45. Scale bars represent 1 µm. b. Exponential phase *B. abortus* carrying either the pBBR-*p*$_{ada}$-*ada-gfp* reporter system or its mutated version (*ada*^{C38A}) were cultured in rich medium (TSB) and exposed to various doses of MMS for 1 or 6 h. Mean fluorescence intensities (MFI) were calculated for n = 150 bacteria in each condition. Scheffe statistical analyses (one way Anova) were performed with $p < 0.05$ (*) and $p < 0.001$ (***), clearly showing that both time of exposure and dose of alkylating agent impact the response of the reporter system. Source data are provided as a Source Data file. c. The two versions of the reporter system were also tested in the WT and Δ*ada1* Δ*ada2* *B. abortus* backgrounds exposed to 2.5 mM MMS for 1 h. Student's *t* test revealed that there is no statistical difference in the responses between the two genetic backgrounds ($p > 0.05$, NS). Error bars represent standard deviation from the mean. Source data are provided as a Source Data file. d. Representative experiment of the reporter system used during macrophages infection, with n = 60, 60, 60 and 55 bacteria, respectively. Black lines represent median values of MFI. Grey bar plots represent ratio (*ada/ada*^{C38A}) of median MFI for each time point. Source data are provided as a Source Data file.

Supplementary Figure 4

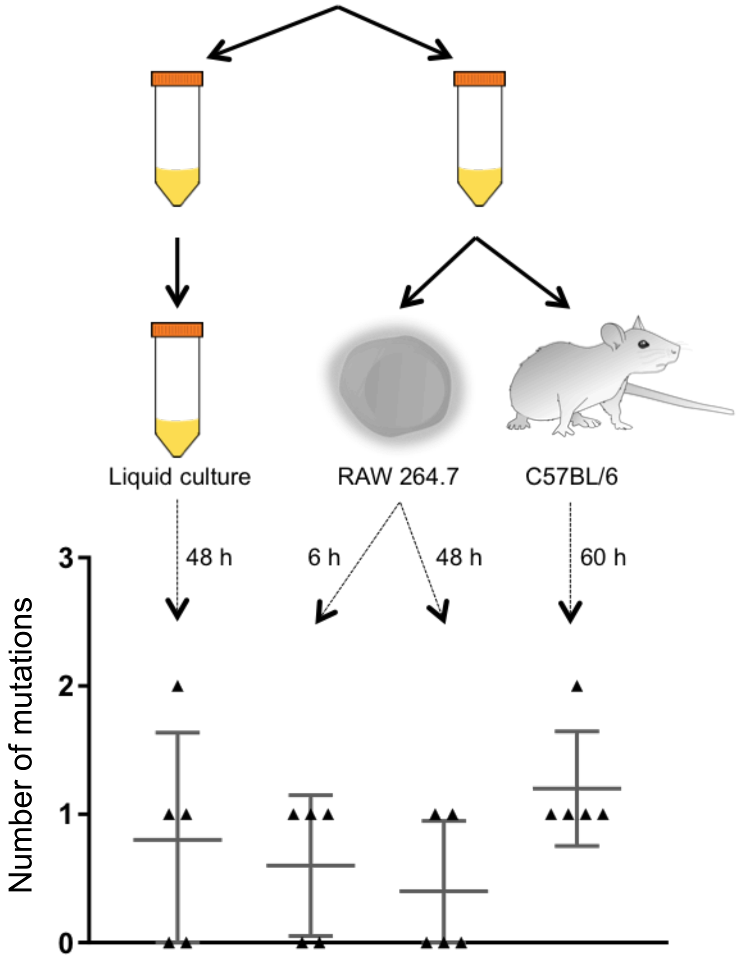

Supplementary Figure 4. Number of mutations occurring after infection and liquid cultures. Whole genome sequencings were performed on liquid cultures resulting from colonies originating from (1) liquid cultures diluted twice in the course of 48 h, (2) bacteria recovered after 6 and 48 h post infection in RAW 264.7 macrophages and (3) bacteria recovered from mice spleen after 60 h of infection (n = 5 for each condition). Microsatellites and positions with less than 10 reads were excluded. All mutations occurred in the *btaE* gene (BAB1_0069), except one in the *cls* gene (BAB2_1021, cardiolipin synthase-like gene) after mice infection. A one-way Anova followed by a Tukey's statistical analysis was performed with $p > 0.05$ (NS).

Supplementary Figure 5

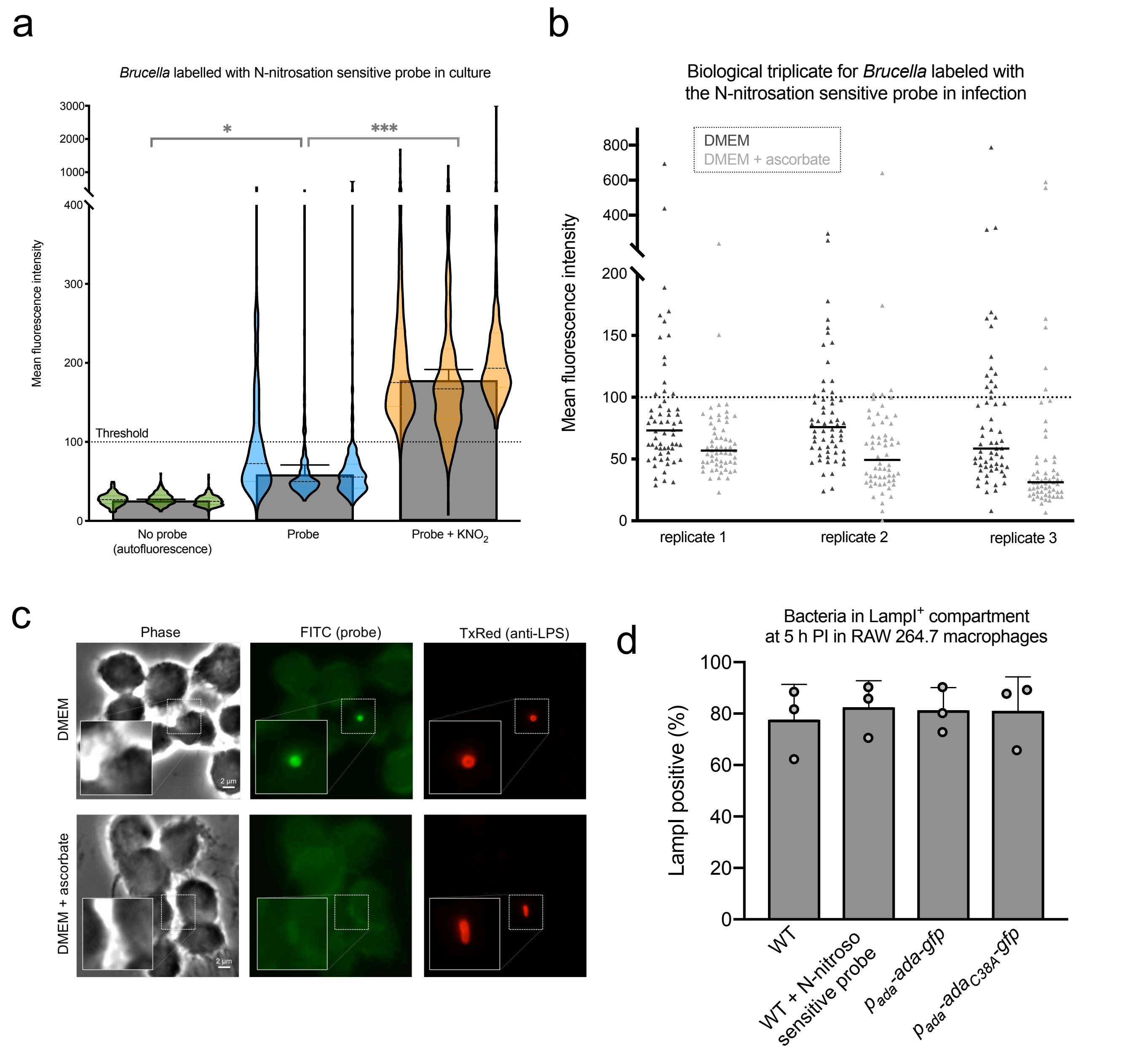

Supplementary Figure 5. *Brucella* labeled with N-nitrosation sensitive probe. a. The mean fluorescence intensities (MFI) of bacteria (FITC channel) were measured after 1 hour of incubation in PBS supplemented (in orange) or not (in blue) with KNO2, a N-nitrosating agent. MFI were also measured for non-labeled bacteria (in green) in order to evaluate B. abortus autofluorescence (No probe). Experiments were done in biological triplicates (violin plots) and median values of MFI were plotted (bar plots). Error bars represent standard deviations. The number of bacteria analyzed in this study were 152, 333, 556 for non-labeled bacteria; 155, 260, 246 for labeled bacteria; and 430, 402, 496 for labeled bacteria subjected to KNO2. A one-way ANOVA followed by a Tukey's statistical analyzes were performed on bar plots with $p < 0.05$ (*) and $p < 0.001$ (***). Source data are provided as a Source Data file. b. Data at the single cell level for the biological triplicate of labeled bacteria ($n = 60$ bacteria) inside host macrophages cultured with DMEM (dark grey) or DMEM supplemented with ascorbate (light grey). The threshold of positive fluorescent signal was set at MFI = 100 based on data obtained in culture. Source data are provided as a Source Data file. c. Images of labeled bacteria inside host macrophages cultures in DMEM with or without ascorbate. d. LampI labeling of RAW 264.7 macrophages to evaluate the proportion of bacteria in eBCV at 5 h post infection (PI). The number of bacteria analyzed in this study were 170, 188, 346 for WT bacteria; 167, 149, 227 for labeled bacteria; 158, 85, 361 for bacteria carrying the functional reporter system; and 164, 86, 253 for bacteria carrying the non-functional reporter system. Source data are provided as a Source Data file.

Supplementary Figure 6

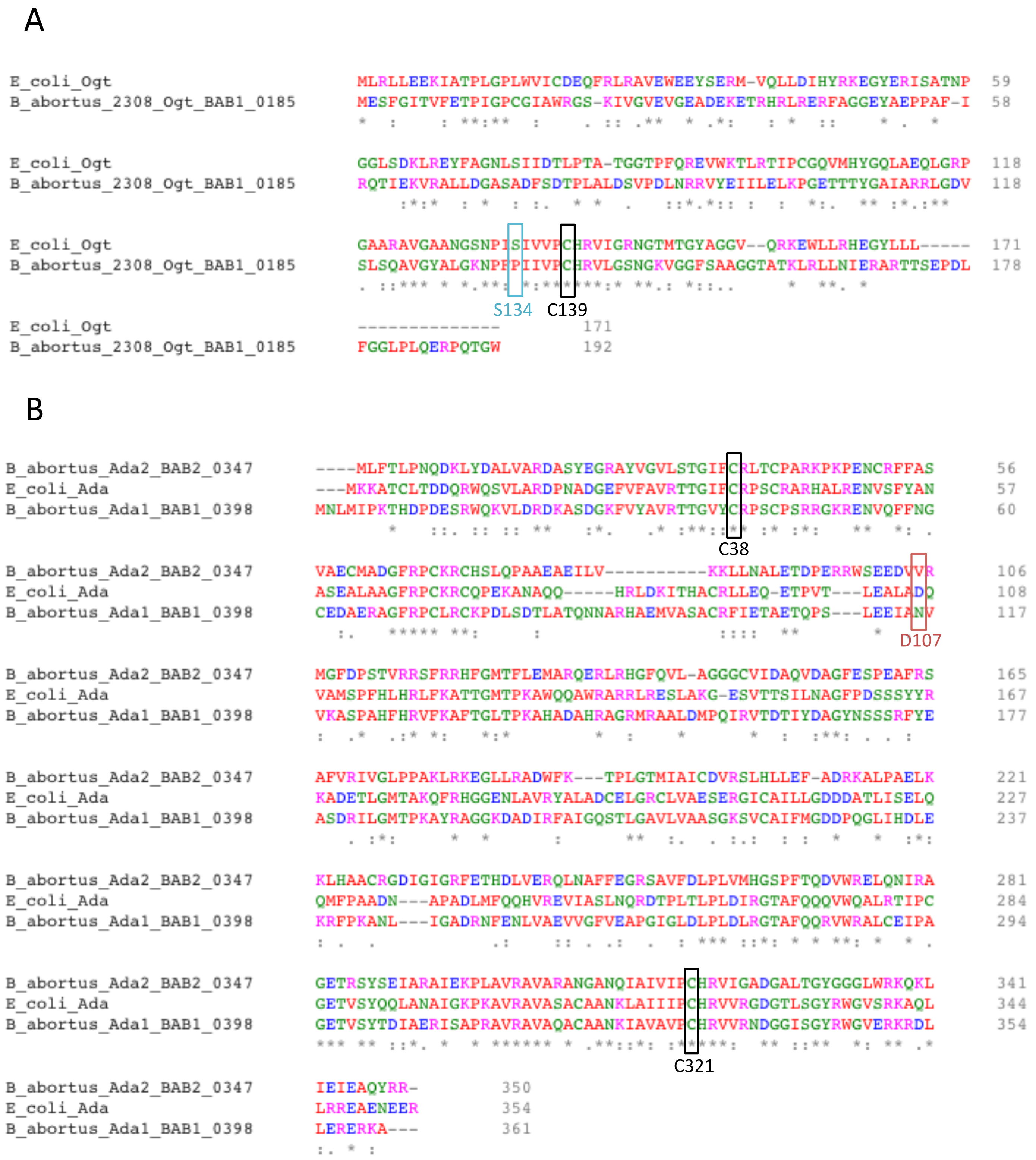

Supplementary Figure 6. Sequence alignments for **A)** *ogt* and **B)** *ada* in *E. coli* K12 and *B. abortus* 2308. Clustal Omega (https://www.ebi.ac.uk/Tools/msa/clustalo/) was used to align amino acid sequences. Black boxes indicate catalytic sites in *E. coli* (C39 and C321 for O^6-methylguanine and O^4-methylthymine repair and C38 for methylphosphotriester capture). The blue box corresponds to a position that confers broader substrate specificity to Ogt, if mutated into a proline, as in *B. abortus*. The red box indicates a position which could be responsible for the absence of a functional adaptive response in *B. abortus*.

Supplementary Figure 7

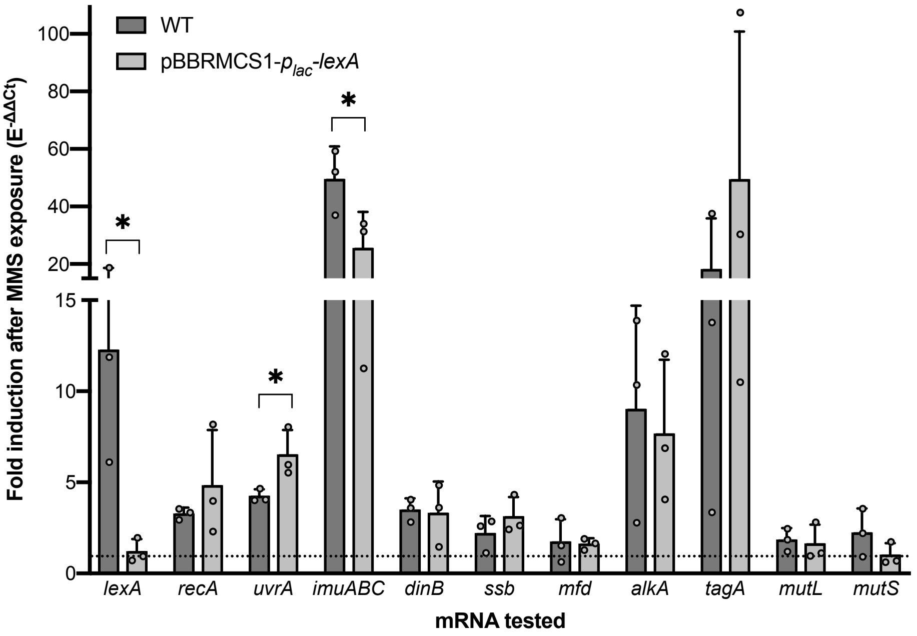

Supplementary Figure 7. Comparison of gene fold induction after 2.5 mM MMS exposure for 5 in the WT or *lexA* overexpression background (pBBRMCS1-p_{lac}-*lexA*), compared to non-stressed bacteria in the same backgrounds. Note that *recA*, *imuABC* (coding for an error-prone DNA polymerase), *dinB* (coding for the error-prone DNA polymerase IV), *ssb* (coding for the single strand binding protein) and *mfd* (coding for the transcription-repair coupling factor) were included in this assay because they all present a TGTTC-N6-TGTTCT LexA-binding motif (based on Erill *et al.*, 2004, Nucleic Acids Res.) upstream of their coding sequence, with one substitution allowed in RSAT (Van Helden, 2003, Nucleic Acids Res.). Experiments were repeated three times. Error bars represent standard deviation from the mean. Student's paired *t* test was performed with $p > 0.05$ (not significant, not indicated) and $p < 0.05$ (*). Source data are provided as a Source Data file.

Supplementary Figure 8

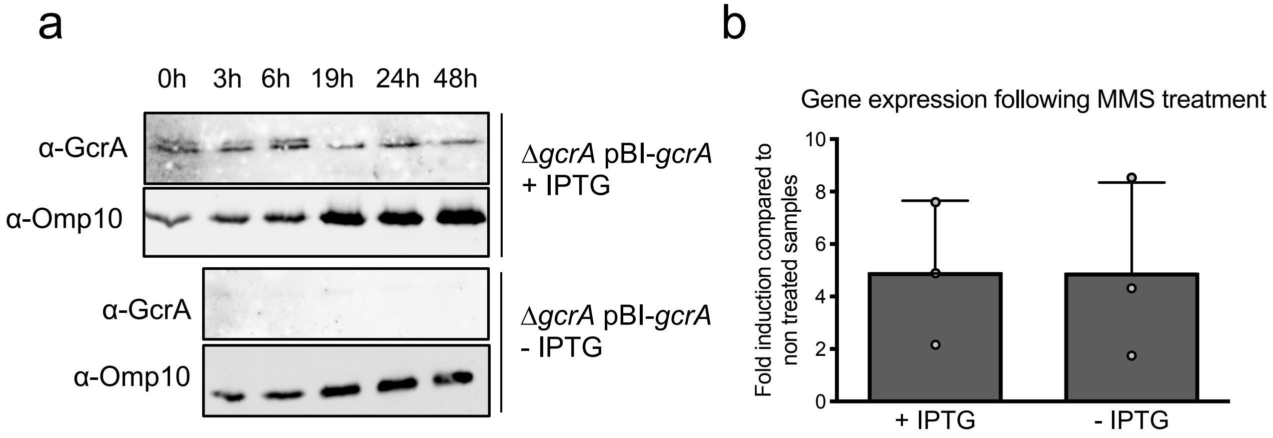

Supplementary Figure 8. GcrA depleted strain and *lexA* expression. **a.** Western blot against GcrA in the presence or absence of IPTG, with different timings post IPTG removal. Omp10 was used as loading control, as it was not amongst GcrA targets according to ChIP-seq experiment. Source data are provided as a Source Data file. **b.** RT-qPCR data of *lexA* gene expression in a GcrA depletion background in presence (+) or absence (-) of IPTG, after 2.5 mM MMS exposure for 5 h, compared to bacteria not exposed to MMS. Experiments were repeated three times. Error bars represent standard deviation from the mean. Student's *t* test was performed with $p > 0.05$ (not significant). Source data are provided as a Source Data file.

Supplementary Figure 9

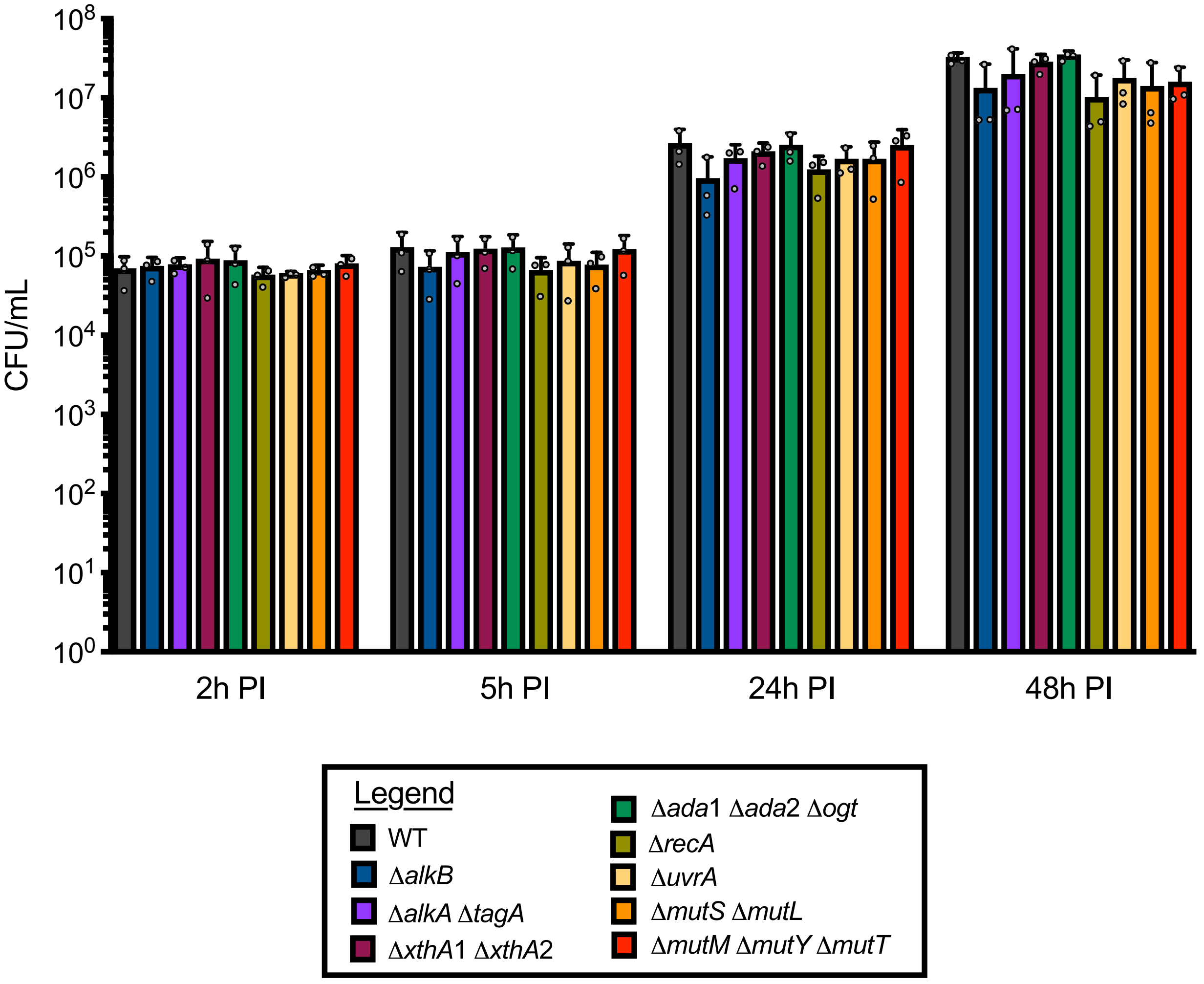

Supplementary Figure 9. Infection of RAW 264.7 macrophages with deletion strain. Colony forming units were counted after 2, 5, 24 and 48 h post infection. Error bars represent standard deviations (n = 3). A Scheffe statistical analysis (one way Anova) reveals that, in this model, none of the tested strains were attenuated in infection ($p > 0.05$, non significant). Source data are provided as a Source Data file.

Supplementary Note 1

A superfolder *gfp* coding sequence, with a *XhoI* sequence after the start codon and a *PstI* sequence (see capital letters) after the stop codon, was adapted to fit the codon usage of *B. abortus* 2308 (http://www.kazusa.or.jp/codon/) and ordered as gBlocks gene fragment (Integrated DNA Technologies):

taatgCTCGAGtcgaagggcgaagaactgttcaccggcgtggtgccgatcctggtggaactggatggcgatgtgaatg
gccataagttctccgtgcgcggcgaaggcgaaggcgatgccaccaatggcaagctgaccctgaagttcatctgcaccaccggcaag
ctgccggtgccgtggccgaccctggtgaccaccctgacctatggcgtgcagtgcttctcgcgctatccggatcatatgaagcgccatg
atttcttcaagtcggccatgccggaaggctatgtgcaggaacgcaccatctcgttcaaggatgatggcacctataagacccgcgccga
agtgaagttcgaaggcgataccctggtgaatcgcatcgaactgaagggcatcgatttcaaggaagatggcaatatcctgggccataag
ctggaatataatttcaattcgcataatgtgtatatcaccgccgataagcagaagaatggcatcaaggccaatttcaagatccgccataatg
tggaagatggctcggtgcagctggccgatcattatcagcagaataccccgatcggcgatggcccggtgctgctgccggataatcattat
ctgtcgacccagtcggtgctgtcgaaggatccgaatgaaaagcgcgatcatatggtgctgctggaattcgtgaccgccgccggcatca
cccatggcatggatgaactgtataagtgaCTGCAGaaaa

Supplementary Note 2

Characterisation of N-nitrosation sensitive probe:

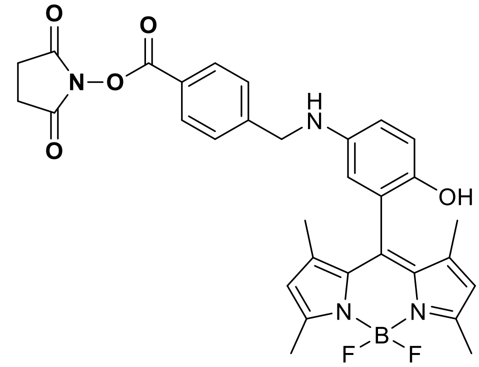

^{1}H NMR ($CDCl_3$, 400 MHz) δ ppm 1.51 (s, 6H, CH_3), 2.52 (s, 6H, CH_3), 2.89 (bs, 4H, CH_2NCO), 4.37 (bs, 2H, Bn), 5.98 (bs, 2H), 6.34 (d, *J* = 2.8 Hz, 1H), 6.65 (dd, *J* = 8.8, 2.8 Hz, 1H), 6.82 (d, *J* = 8.4, 1H), 7.45 (d, *J* = 8.8, 2H), 8.05 (*J* = 8.8, 2H).

^{13}C NMR ($CDCl_3$, 100 MHz) δ 13.78 (CH_3), 14.69 (CH_3), 25.75(CH_2N), 48.55 (Bn), 113.06, 116.68, 117.80, 121.53, 121.77, 124.06, 127.63, 128.87, 131.01, 131.32, 136.01, 142.18, 143.44, 144.67, 147.23, 156.32, 161.72 (CO), 169.36 (NCO).

HRMS: (ESI+-MS, m/z) calculated for $C_{28}H_{28}BF_2N_3O_3$ $[M+H]^+$: 587.2277, found: 504.2271.

Melting point: 114-116 ^{o}C

IR: 3370 (N-H), 2920 (C-H), 1732 (C=O), 1542.8, 1504 $(C=C)_{Ar}$, 1190 (C-O), 1306, 810.5 cm^{-1}.

Supplementary Note 3

Protocol to analyse mean fluorescence intensity of *B. abortus* with MicrobeJ

1) Start ImageJ
2) Click on File/Import/Image sequence and choose the file containing the images (TIFF) corresponding to the anti-LPS signal (12G12) (or phase contrast for bacteria on pads)
3) In sequence Options, click on OK (scale images 100%; sort names numerically)
4) Click on Image/Lookup tables/Red (if secondary antibody of IF was TxRed-conjugated)
5) Click on File/Import/Image sequence and choose the file containing the images (same names as in point 2!) corresponding to fluorescent signal of interest (e.g. reporter system with GFP)
6) In sequence Options, click on OK (scale images 100%; sort names numerically)
7) Click on Image/Lookup tables/Green
8) Go to Plugins/MicrobeJ/MicrobeJ
9) In the window Images, change "image" to "channels" and attribute TxRed to Ch. 1 and GFP to Ch. 2, then click on 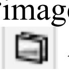 to fuse them.
10) In the window Bacteria, select "channel 1: Dark" for intracellular bacteria (and Bright for bacteria on pads), then roll your mouse on the play (>) button at the bottom, to make it appear as a >> and click on it. This will trigger the detection of bacteria on all images.
11) Click on the pencil to redo a detection of bacteria that will allow the modification of selections. A new window (Experiment Editor) will appear and you will get more tools in your toolbox):

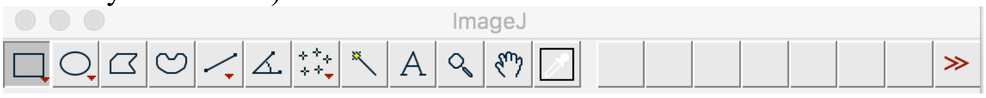

becomes

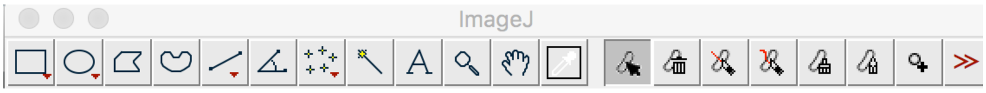

12) Start making modifications/corrections of bacterial detection in the window DUP Hyperstack with the new tools on the right of ImageJ toolbox (play with them to learn to use then, it's easy). When doing so, you should only look in the first colour channel (Red) to avoid bias. When making corrections, you will see that you also create "rubbish selections" of unwanted pixels. To erase them, you can use the bin tool, but you risk to also touch a good selection, so I don't recommend it. Instead, go in the window Experiment Editor and click on one bacterium. Its selection will appear in blue in the DUPH. If you want to erase it, click on the bin in the ExpEd bottom.
13) Before finishing, check all bacteria with the Magnifying glass tool in the ImageJ window while clicking on a bacterium (then click "arrow down" for the next one) in ExpEd to see if the selections are good and that you did not forget a small selected "rubbish".
14) In the ExpEd, select all your bacteria (ctlr + A), than click on the small "V" sign (note that sometimes this step is not needed).
15) Click on the 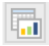 button to open the Result window.
16) Click on Bacteria under Experiment to make the list appear.
17) Right click on the PROFILE_MED column and click on ch2 to make the same column appear for the green signal.

18) Right click on the INTENSITY column/ch2/mean to make a new column appear. This column gives you the mean fluorescence intensity for one bacterium after removing the background intensity!
19) Right click on the columns that do not interest you to "Hide col(s)" and simplify your table.
20) Save your data by clicking on the "save A+" button.
21) If your computer is set up in English, you can export your data to excel! If not, good luck and have fun copying everything manually… ;)
22) In the excel file, create columns:

**mean (fluo)**	**Bckgd**	**Result**		**Bckgd 1**	**Bckgd 2**	**Bckgd 3**
= data	= mean (x;y;z)	= mean - bckgd		x	y	z

23) For each bacteria, determine 3 background values manually by clicking on the bacteria (in ExpEd or result) while looking at them with the magnifying glass and using the SECOND channel (green in this case), then in the ImageJ toolbar, click on >> and select Pixel Inspector. Click in an area very close (but avoid directly touching) to your bacteria to make a square appear and copy the central pixel value as x, then click on an other side of your bacterium to get the value for y, then z.
24) When you have done so for all your bacteria (I usually stop after n = 60), you can copy-paste the results column as your final results.

NOTE: the **mean_c** (= mean fluo – background) values can be used only in the case of bacteria on PBS-agarose pads (with even background), but not inside cells.
