# Supplementary material for: Occurrence and repair of alkylating stress in the intracellular pathogen Brucella abortus
Source: Nat Commun. 2019 Oct 24;10:4847. doi: 10.1038/s41467-019-12516-8 (PMC6813329; doi:10.1038/s41467-019-12516-8)
Supplement: Supplementary file 3 — Description of Additional Supplementary Files [file 41467_2019_12516_MOESM3_ESM.pdf]

Description of Additional Supplementary Files

File Name: Supplementary Data 1

Description: List of primers, strains and plasmids used during this study.
