# Supplementary material for: Occurrence and repair of alkylating stress in the intracellular pathogen Brucella abortus
Source: Nat Commun. 2019 Oct 24;10:4847. doi: 10.1038/s41467-019-12516-8 (PMC6813329; doi:10.1038/s41467-019-12516-8)
Supplement: Supplementary file 5 — Reporting Summary [file 41467_2019_12516_MOESM5_ESM.pdf]

nature research

Corresponding author(s): XDB

Last updated by author(s): Jun 18, 2019

Reporting Summary

Nature Research wishes to improve the reproducibility of the work that we publish. This form provides structure for consistency and transparency in reporting. For further information on Nature Research policies, see Authors & Referees and the Editorial Policy Checklist.

Statistics

For all statistical analyses, confirm that the following items are present in the figure legend, table legend, main text, or Methods section.

n/a	Confirmed	
☐	☒	The exact sample size (*n*) for each experimental group/condition, given as a discrete number and unit of measurement
☐	☒	A statement on whether measurements were taken from distinct samples or whether the same sample was measured repeatedly
☐	☒	The statistical test(s) used AND whether they are one- or two-sided<br>*Only common tests should be described solely by name; describe more complex techniques in the Methods section.*
☒	☐	A description of all covariates tested
☒	☐	A description of any assumptions or corrections, such as tests of normality and adjustment for multiple comparisons
☐	☒	A full description of the statistical parameters including central tendency (e.g. means) or other basic estimates (e.g. regression coefficient) AND variation (e.g. standard deviation) or associated estimates of uncertainty (e.g. confidence intervals)
☐	☒	For null hypothesis testing, the test statistic (e.g. *F*, *t*, *r*) with confidence intervals, effect sizes, degrees of freedom and *P* value noted<br>*Give P values as exact values whenever suitable.*
☒	☐	For Bayesian analysis, information on the choice of priors and Markov chain Monte Carlo settings
☒	☐	For hierarchical and complex designs, identification of the appropriate level for tests and full reporting of outcomes
☒	☐	Estimates of effect sizes (e.g. Cohen's *d*, Pearson's *r*), indicating how they were calculated

Our web collection on statistics for biologists contains articles on many of the points above.

Software and code

Policy information about availability of computer code

Data collection	no software was used
Data analysis	no software was used

For manuscripts utilizing custom algorithms or software that are central to the research but not yet described in published literature, software must be made available to editors/reviewers. We strongly encourage code deposition in a community repository (e.g. GitHub). See the Nature Research guidelines for submitting code & software for further information.

Data

Policy information about availability of data

All manuscripts must include a data availability statement. This statement should provide the following information, where applicable:

- Accession codes, unique identifiers, or web links for publicly available datasets
- A list of figures that have associated raw data
- A description of any restrictions on data availability

All data supporting the findings of this study are available from public repositories or from the corresponding author on reasonable request.

Field-specific reporting

Please select the one below that is the best fit for your research. If you are not sure, read the appropriate sections before making your selection.

☒ Life sciences ☐ Behavioural & social sciences ☐ Ecological, evolutionary & environmental sciences

For a reference copy of the document with all sections, see nature.com/documents/nr-reporting-summary-flat.pdf

Life sciences study design

All studies must disclose on these points even when the disclosure is negative.

Sample size	no sample size calculation was performed, sample size was determined according to previous experience in the field, to detect differences that are statistically significant
Data exclusions	No data were excluded from the analyses
Replication	All experiments were reproduced at least three times with independent biological samples, except for mice experiments, that were repeated twice and gave each time statistically relevant differences with groups of 8-9 animals (Fig. 8). The differences of interest were huge, and we thus decided to not sacrify more animals.
Randomization	Randomization was not relevant to our study since samples are different in nature, e.g. a mutant and a wild type strain, or a mRNA or another.
Blinding	Blinding was not possible, due to the constraints imposed by the work in a Biosafety Level 3 facility, with a limited access.

Reporting for specific materials, systems and methods

We require information from authors about some types of materials, experimental systems and methods used in many studies. Here, indicate whether each material, system or method listed is relevant to your study. If you are not sure if a list item applies to your research, read the appropriate section before selecting a response.

Materials & experimental systems

n/a	Involved in the study	
☐	☒	Antibodies
☐	☒	Eukaryotic cell lines
☒	☐	Palaeontology
☐	☒	Animals and other organisms
☒	☐	Human research participants
☒	☐	Clinical data

Methods

n/a	Involved in the study	
☐	☒	ChIP-seq
☒	☐	Flow cytometry
☒	☐	MRI-based neuroimaging

Antibodies

Antibodies used	homemade anti-Brucella rabbit polyclonal antibodies (ref 27), anti-LampI rat antibodies (1D4B, Developmental Studies Hybridoma Bank, University of Iowa), goat anti-rabbit antibodies coupled to Pacific Blue (Invitrogen, cat. no. P10994), goat anti-rat antibodies coupled to Alexa Fluor 647 (Invitrogen, cat. no. A21247), monoclonal anti-O chain A76-12G12 (ref 77), goat anti-mouse secondary antibodies coupled to Texas Red (1:500) (Invitrogen, cat. no. T862)
Validation	Validation of these antibodies were performed in previous works, as well as negative controls when possible (e.g. in the basence of primary antibody to control for secondary antibodies specificity)

Eukaryotic cell lines

Policy information about cell lines

Cell line source(s)	RAW 264.7 macrophages were purchased from ATCC
Authentication	The cell line was not authenticated, but we are working with this cell line since many years
Mycoplasma contamination	The cell line was not tested recently for Mycoplasma contamination
Commonly misidentified lines (See ICLAC register)	not applicable

Animals and other organisms

Policy information about studies involving animals; ARRIVE guidelines recommended for reporting animal research

Laboratory animals	Mouse (Mus musculus), strain C57BL/6, 10 to 12 weeks old, only females for the first experiment, and a mix of males and females for the second experiment (both shown in Fig. 8). For genome sequencing after infection (Fig. S4), 10 to 12 weeks old females were infected.
Wild animals	*Provide details on animals observed in or captured in the field; report species, sex and age where possible. Describe how animals*

Wild animals	*were caught and transported and what happened to captive animals after the study (if killed, explain why and describe method; if released, say where and when) OR state that the study did not involve wild animals.*
Field-collected samples	*For laboratory work with field-collected samples, describe all relevant parameters such as housing, maintenance, temperature, photoperiod and end-of-experiment protocol OR state that the study did not involve samples collected from the field.*
Ethics oversight	*Identify the organization(s) that approved or provided guidance on the study protocol, OR state that no ethical approval or guidance was required and explain why not.*

Note that full information on the approval of the study protocol must also be provided in the manuscript.

ChIP-seq

Data deposition

☒ Confirm that both raw and final processed data have been deposited in a public database such as GEO.

☒ Confirm that you have deposited or provided access to graph files (e.g. BED files) for the called peaks.

Data access links<br>*May remain private before publication.*	https://figshare.com/s/0e580305b65f67619d36
Files in database submission	For each chromosome, a .gb file and a txt file with the number of reads aligned at each position of the sequence in the .gb file
Genome browser session<br>(e.g. UCSC)	Data indicated above can be visualized with the freely available Artemis software

Methodology

Replicates	Data were generated for only one sample
Sequencing depth	In the ChIP-seq experiment, there were 1.19 millions aligned reads, read length was 50 (single end). Brucella genome (about 3.29 Mb) contains >98% non-repeated sequences
Antibodies	We used a home-made rabbit polyclonal antibody to purified GcrA-His6 recombinant protein. Specificity of the antibodies were checked by western blotting (see Source data file).
Peak calling parameters	The distribution of the number of reads aligned at each position of the genome was used to evaluate mean and standard deviation (here called Z) over a window of 1 million base pairs. Peaks were defined as regions in which the number of reads aligned to the genome sequence was 4Z above the average.
Data quality	In theory, the method with 4 standard deviations (4Z) above the average generates a background of 0.0032% of false positive values
Software	ChIP-seq data were analyzed with Excel (bacterial genomes are small enough)
